# Microbial population dynamics during long-term sludge adaptation of thermophilic and mesophilic sequencing batch digesters treating sewage fine sieved fraction at varying organic loading rates

**DOI:** 10.1186/s13068-015-0355-3

**Published:** 2015-10-21

**Authors:** Dara S. M. Ghasimi, Yu Tao, Merle de Kreuk, Marcel H. Zandvoort, Jules B. van Lier

**Affiliations:** Sanitary Engineering Section, Department of Water Management, Faculty of Civil Engineering and Geosciences, Delft University of Technology, Stevinweg 1, 2628 CN Delft, The Netherlands; Department of Chemical Engineering, Imperial College London, South Kensington Campus, London, SW7 2AZ the UK; Waternet, Korte Ouderkerkerdijk 7, P.O. Box 94370, 1090 GJ Amsterdam, The Netherlands

**Keywords:** Anaerobic digestion, Fine sieved fraction (FSF), Cellulose, Volatile fatty acids (VFAs), Adaptation, Microbial community

## Abstract

**Background:**

In this research, the feasibility of, and population dynamics in, one-step anaerobic sequencing batch reactor systems treating the fine sieved fraction (FSF) from raw municipal wastewater was studied under thermophilic (55 °C) and mesophilic (35 °C) conditions. FSF was sequestered from raw municipal wastewater, in the Netherlands, using a rotating belt filter (mesh size 350 micron). FSF is a heterogeneous substrate that mainly consists of fibres originating from toilet paper and thus contains a high cellulosic fraction (60–80 % of total solids content), regarded as an energy-rich material.

**Results:**

Results of the 656-day fed-batch operation clearly showed that thermophilic digestion was more stable, applying high organic loading rates (OLR) up to 22 kg COD/(m^3^ day). In contrast, the mesophilic digester already failed applying an OLR of 5.5 kg COD/(m^3^ day), indicated by a drop in pH and increase in volatile fatty acids (VFAs). The observed viscosity values of the mesophilic sludge were more than tenfold higher than the thermophilic sludge. 454-pyrosequencing of eight mesophilic and eight thermophilic biomass samples revealed that *Bacteroides* and aceticlastic methanogen *Methanosaeta* were the dominant genera in the mesophilic digester, whereas OP9 lineages, *Clostridium* and the hydrogenotrophic methanogen *Methanothermobacter* dominated the thermophilic one.

**Conclusions:**

Our study suggests that applying thermophilic conditions for FSF digestion would result in a higher biogas production rate and/or a smaller required reactor volume, comparing to mesophilic conditions.

**Electronic supplementary material:**

The online version of this article (doi:10.1186/s13068-015-0355-3) contains supplementary material, which is available to authorised users.

## Background

Cellulose makes up about 30–50 % of the suspended solids in the sewage of western countries [1] and it usually enters aerobic sewage treatment plants, adding significant costs due to difficulties in aerobic degradation [2]. At sewage treatment plant (STP) Blaricum (Netherlands), the sewage flow is directed through a fine sieve with a mesh size of 350 µm. This sieve is implemented as a compact alternative to primary clarification to separate suspended solids from sewage prior to biological nutrient removal. The fine sieved fraction (FSF) is a heterogeneous substrate with high bio-energy potential. It mainly consists of partly disrupted toilet paper and cellulose accounts for 79 % of the total mass and 84 % of the organic mass according to a thermographic analysis [2]. Because FSF has a high dry solids concentration (20–30 %), a straightforward method to stabilise it is by dry anaerobic digestion (AD) [3].

Efficient AD of solids can proceed either under mesophilic (30–40 °C) or thermophilic (50–60 °C) conditions [4–6]. Many previous studies indicate that, compared to mesophilic AD processes, thermophilic AD generally accepts a higher organic loading rate, more efficient degradation of organic matters [7, 8], higher biogas production efficiency [9–12] and better sludge dewaterability [13]. It is hypothesised that microbial-ecology-driven factors play vital roles differentiating the performance in the abovementioned aspects between thermophilic and mesophilic dry digesters. However, it still remains unknown about how and to which extent temperature can shape microbial communities during long-term dry anaerobic digestion.

In this study, two parallel anaerobic digesters were operated for over 2 years under 55 and 35 °C. The two digesters were fed with the FSF from STP Blaricum as a sole substrate in batch mode. The overall aim was to seek for insights into how the thermophilic and mesophilic communities will adapt and respond to an increasing organic loading rates (OLRs) of FSF. The digesters performance such as biogas production, volatile fatty acids (VFAs) and sludge viscosity were followed to indicate the possible functional dynamics of the communities along with their structural variation.

## Results and discussion

### Composition and surface architecture of FSF

**Fig. 1:**
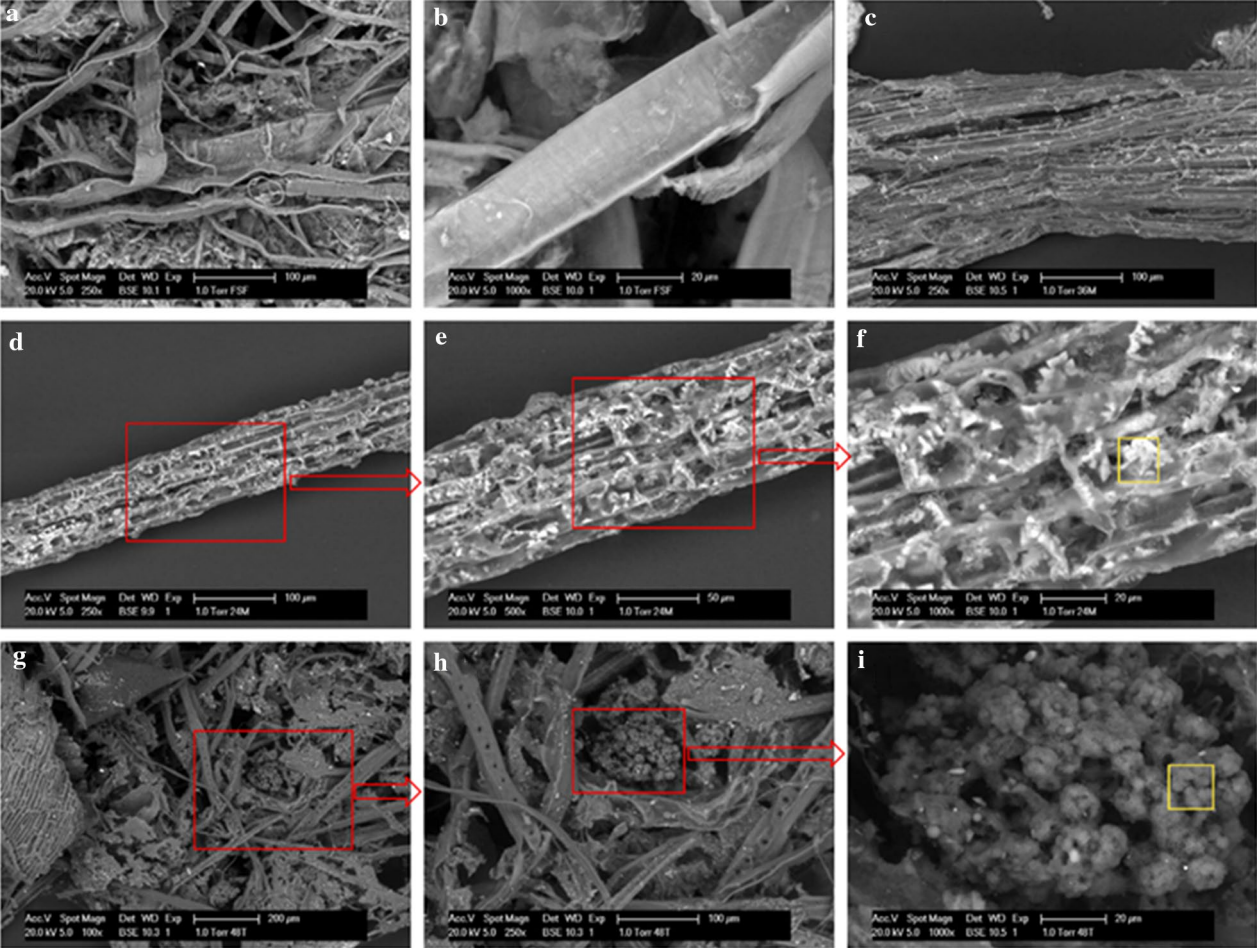
Scanning electron microscopic photograph of raw FSF matrix (**a**), smooth fibre in the raw FSF (**b**), partially fractured FSF after 36 h in the mesophilic digester (**c**), step-by-step magnified sample from the mesophilic digester after 24 h digestion (**d**–**f**), bacterial clusters within the digested FSF matrix sampled from the thermophilic digester after 48 h digestion (**g**–**i**). The areas in *red rectangles* (**d**, **g**) represent the zoom-in areas of the subsequent figures (**e**, **h**), respectively. The areas in *yellow rectangles* (**f**, **i**) were the scanning location for the EDX analysis (results shown in Additional file 1: Table S1)

Composition and surface morphology of solid wastes are often considered determining factors to hydrolysing processes. Therefore, it is important to characterise the FSF in detail. First of all, environmental scan electron microscopy and energy dispersive X-ray (ESEM-EDX) analysis revealed carbon (60–64 %) and oxygen (20–27 %) as major elements in raw FSF materials [3], while a previous thermographic test showed that cellulose accounted for 79 % of the total mass and 84 % of the organic dry mass of raw FSF [2]. Secondly, the FSF that were collected at the different times showed a great variation in composition during the different seasons of the year due to the inconsistency of composition in the raw domestic wastewater [3]. In addition, all the FSF samples showed a high heterogeneity (Fig. 1a). Based on the above and our experimental BMP data ([3], submitted manuscript), we estimate a varying cellulosic content between 60 and 80 %, depending on mentioned seasonal and influent fluctuations.

Smooth fibres can be seen in the raw FSF (Fig. 1b) and a partially hydrolysed fibre after 36 h in the mesophilic digester can be also seen (Fig. 1c), in which a lot of cracks were present. A series of step-by-step magnified figures were captured to show the hydrolysing process in the mesophilic digester after 24 h, from which gel-like materials and irregular pores can be observed on the surface (Fig. 1d–f). The EDX analysis showed that more than 90 % of the hydrolysed fibre surface was composed of carbon and oxygen (Additional file 1: Table S1). A microbial consortium that was hidden inside the digested FSF matrix was captured in the sample taken from the thermophilic digester after 48 h digestion and very clear coccoid-shape microorganisms can be seen (Fig. 1g–i). The ESEM-EDX results also showed that calcium accounted for almost 2 % in weight on the surface of those coccoid-shape microorganisms (Fig. 1i, Additional file 1: Table S1), which might be derived from the scavenged toilet paper, or from possible inorganic precipitations occurring near the microbial consortia.

### Biogas production and VFA accumulation

**Fig. 2:**
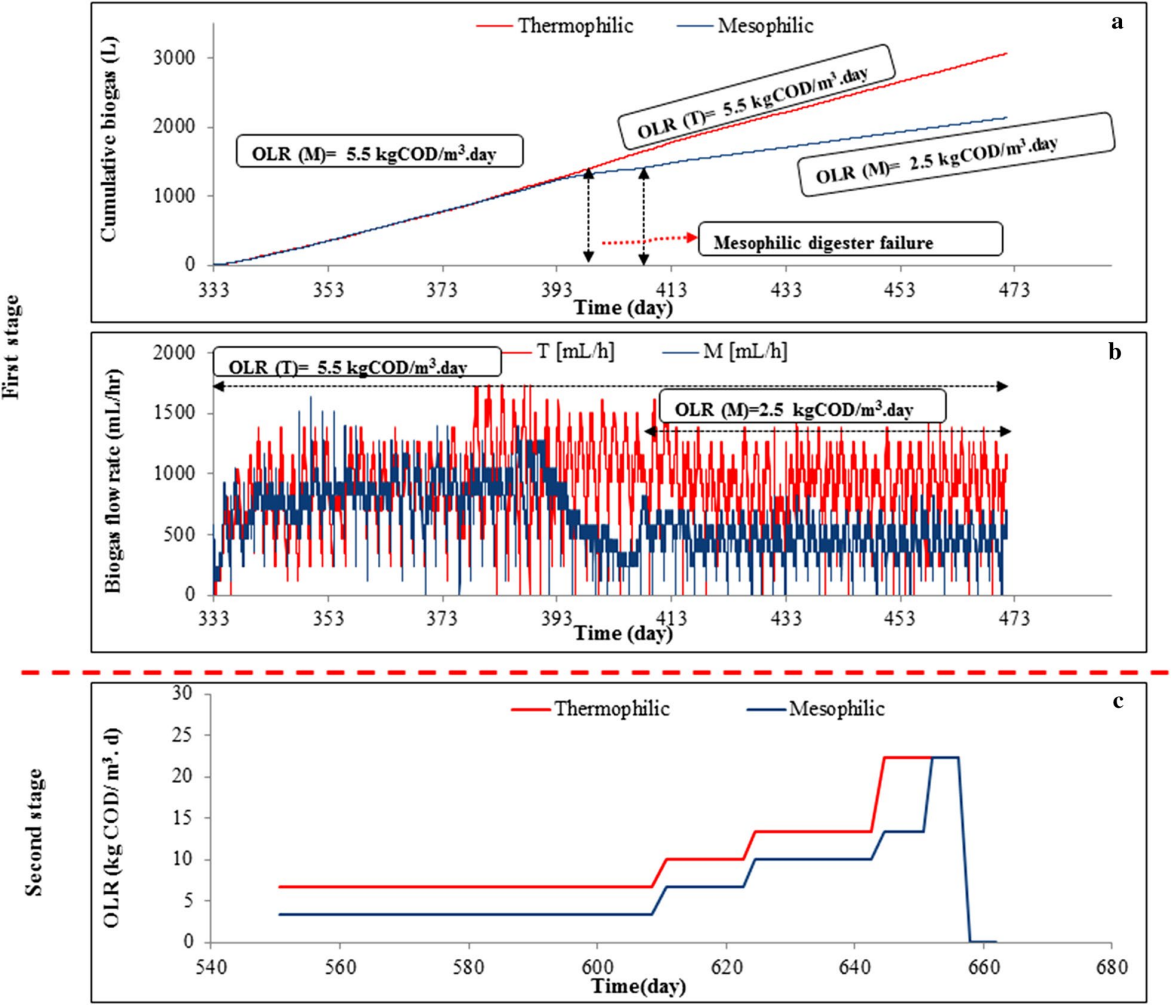
Cumulative biogas production (in L, **a**), biogas flow rate in the thermophilic (T) and mesophilic (M) digesters (in mL/hr, **b**) and OLR in the thermophilic and mesophilic conditions (in kg COD/(m^3^ day), **c**)

**Fig. 3:**
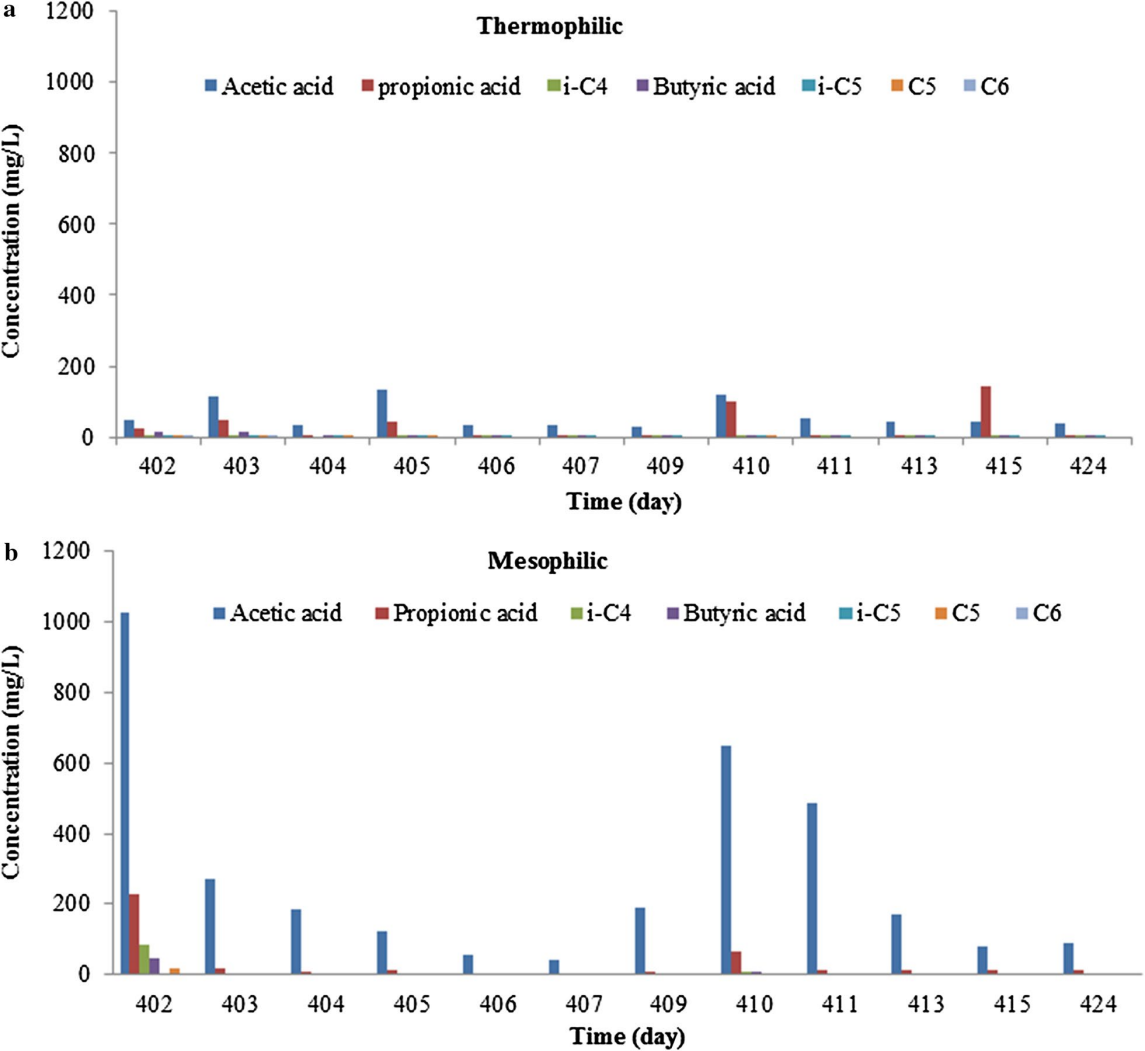
VFA concentrations in the thermophilic reactor (**a**): batch wise feeding occurred at the even days: odd numbered days represent 24 h after feeding, while even days represent 48 h after feeding. The VFA concentrations in the mesophilic reactor (**b**) were measured during recovery and after the batches of FSF at day 407; hereafter even numbered days represent 24 h after feeding, while odd days represent 48 h after feeding

The start-up and adaptation of the mesophilic and thermophilic seed sludge to the FSF has been reported comprehensively in our previous work [3]. A stable process was observed after almost a year of operation. Maximum daily accumulated biogas production rate for the thermophilic and mesophilic digesters was 2.5 L/(L_*reacto*r_∙day), from day 333 till day 393 at an OLR of 5.5 kg COD/(m^3^ day) with FSF chemical oxygen demand (COD) of 350 ± 15 g/kg and total solids (TS) of 25 %, respectively (Fig. 2a). The mesophilic digester became instable from day 393 (Fig. 2a, b), indicated by a rapid decrease in pH and biogas production and increased TS from 4.2 % on day 333 to 6 % on day 396. The lowest registered pH of the digester was 6.3 on day 396 when the FSF digestion process was failing. In order to recover the reactor, feeding was stopped on day 396 and half of the mesophilic sludge in the digester was replaced with excess mesophilic sludge that was collected from the same digester before and stored at room temperature. Mixing both sludges led to a gradual increase in pH and to a drop in VFA concentrations (Fig. 3b). Feeding of the mesophilic digester was restarted on day 407 and the OLR was reduced from 5.5 (SRT of 64 days) to 2.5 kg COD/(m^3^ day) (SRT of 128 days) in order to avoid recurrence of the process instability.

In order to investigate the limits of the loading capacity of both digester systems (Fig. 2c; Table 1), the OLR was increased stepwise from 2.5 to 22 kg COD/(m^3^∙day), leading to a decreased SRT from 128 to 16 days under mesophilic conditions. Therefore, the average biodegradation of the FSF slightly decreased from 53 % at OLR of 2.5 kg COD/(m^3^ day) to 44 % at OLR of 13.5 kg COD/(m^3^ day). Further increase in OLR to 22 kg COD/(m^3^ day) led to an immediate process failure of the mesophilic digestion, biodegradation decreasing to 14 % (Table 1), acetate and propionate accumulating to 3.0 and 2.6 g/L, respectively, and pH dropping to 5.7. It was notable that in this stage, at the applied SRTs, complete conversion of the FSF was not attained in both digesters, which was more strikingly for the mesophilic reactor.

**Table 1:** Average organic loading rates (OLR) and SRTs at different periods and the corresponding biodegradability for both mesophilic and thermophilic conditions

Conditions and operational days	OLR, kg COD/(m^3^∙day)	Applied SRT (day)	Required SRT (day)	AnBD (%)
Mesophilic				
333–393	5.5	60	64	ND
393–550	2.5	157	128	53
550–608	3.4	58	107	54
608–624	6.7	16	53	45
624–644	10.0	20	36	45
644–650	13.5	6	27	44
650–656	22.0	6	16	14
Thermophilic				
333–550	5.5	217	64	60
550–608	6.7	58	53	57
608–624	10.0	16	36	58
624–644	13.5	20	27	47
644–656	22.0	12	16	34

*ND* not detected

For the thermophilic digester, the OLRs were continuously increased from 5.5 kg COD/(m^3^ day) (day 333–550) to 22 kg COD/(m^3^ day) (day 644–656), decreasing the SRT from 64 to 16 days (Fig. 2c; Table 1). When the OLR was increased to 13.5 kg COD/(m^3^ day) the biodegradation reduced from 60 to 47 % (Table 1). A further increase to 22 kg COD/(m^3^ day), further reduced biodegradation to 34 %; at this stage acetate and propionate accumulated to 0.17 and 1.10 g/L, respectively. Nevertheless, the thermophilic digester could still be operated stable and process failure was not experienced. It is notable that, when the feeding to the reactor was paused for several days in the period of applying the highest OLR, the thermophilic reactor produced almost 100 L of CH_4_, which relates to the conversion of about 0.82 kg FSF, assuming a COD content of 350 g/kg. The observed methane peak could be ascribed to the further degradation of accumulated FSF in the digester. When the feeding was restarted, the biogas production rate was fully restored.

Figure 3 shows the VFA concentrations in both thermophilic and mesophilic digesters during recovery of the mesophilic sludge (day 402 to day 424). Restarting the batch wise feeding regime of the mesophilic digester at day 407, resulted in an increased acetate concentration at the end of first two cycles. The reduced OLR of 2.5 kg COD/(m^3^ day), however, was sufficient for stabilising the mesophilic reactor, indicated by decreased acetate concentrations and stable biogas production (Fig. 2b). Within the VFA spectrum produced, acetate and propionate were observed in the highest concentrations under both conditions (Fig. 3). At mesophilic conditions, a gradual pH decrease to 6.3 was observed from day 396 to 402, whereafter the VFA concentrations were measured from day 402 to day 424. Decrease in VFA concentrations (day 402 to day 407) is mainly due to (1) replacing half of content of the mesophilic digester with excess mesophilic sludge collected and stored from the same digester (2) no feeding from day 402 to 407 in order to stabilise the conversion and to recover the sludge (Fig. 2a, b). After 15 days (day 424) the anaerobic digestion was considered stable again.

### Specific methanogenic activity (SMA) of mesophilic and thermophilic biomass

**Fig. 4:**
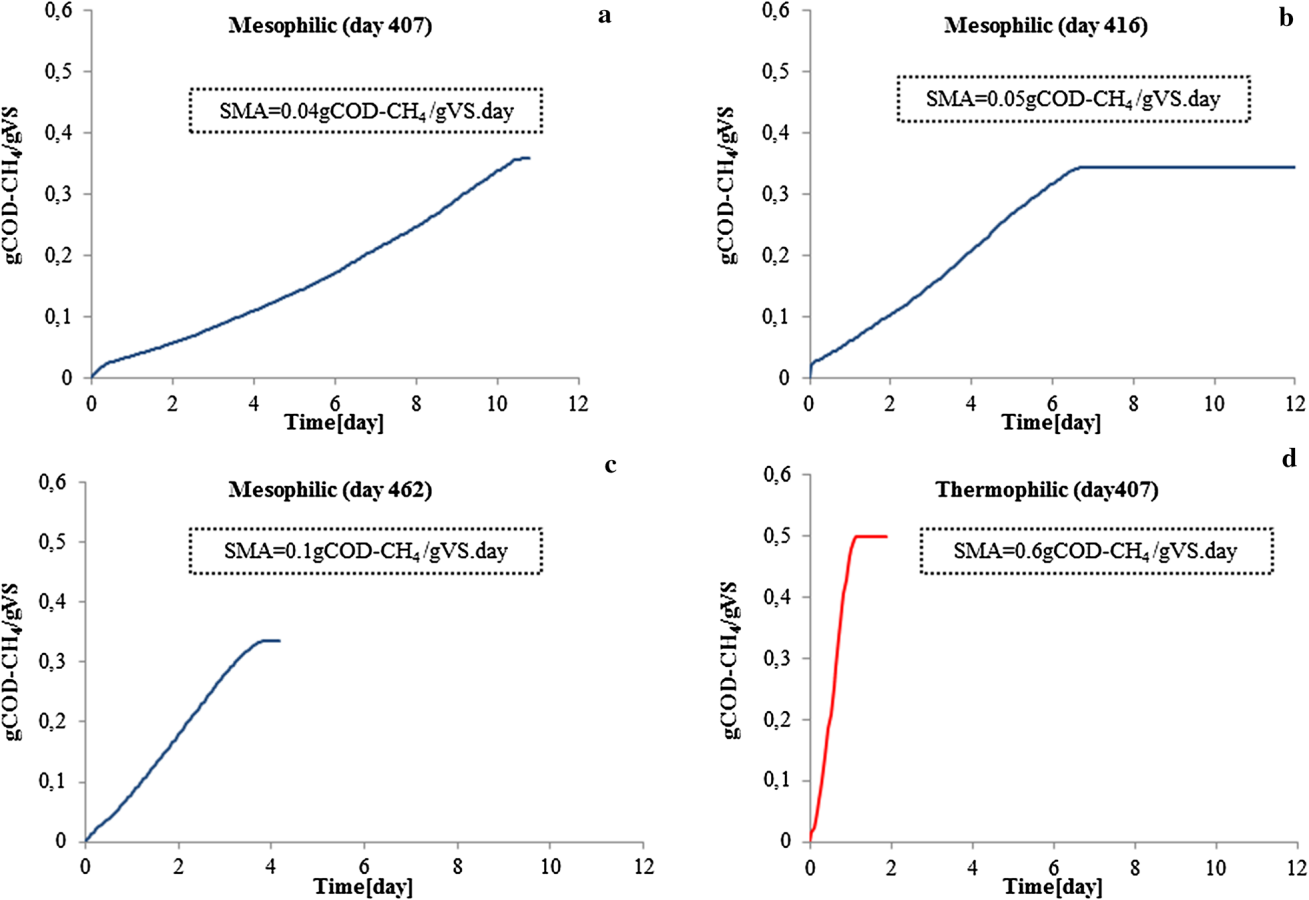
Specific methanogenic activity (SMA) conducted during recovery of the mesophilic digester at OLR of 2.5 kg COD/(m^3^ day) (**a**, **b**, **c**) and of the thermophilic digester as comparison (**d**) at OLR of 5.5 kg COD/(m^3^ day)

To investigate the activity of the methanogens after the mesophilic reactor recovered, four SMA tests were conducted at day 407 (Fig. 4a), day 416 (Fig. 4b) and day 462 (Fig. 4c), after which the reactor was considered to be fully stable again. The SMA of the thermophilic sludge was determined at day 407 only (Fig. 4d). The results showed that the activity of the methanogenic organisms was indeed low at reactor failure, while a long period without VFA accumulation resulted in an increased acetate consumption rate, however, not exceeding 0.1 gCOD-CH_4_/(gVS day) at day 462. This value was considerably lower than the conversion rate measured from the thermophilic sludges, which was 0.6 gCOD-CH_4_/(gVS day) at day 407, higher than most of previously reported values (Table 2). This activity remained the same at the fixed OLR of 5.5 kgCOD/(m^3^ day). The measured high SMA values of the thermophilic sludge are congruent with the observed high reactor stability at increased OLRs, whereas the observed low SMA values of the mesophilic digester agreed with instability and recovery period between day 402 and day 424.

**Table 2:** Comparison of SMA values of this study with previous studies done on (co-)digestion of lignocellulosic wastes

Substrate type	Reactor	Temperature (°C)	SMA	OLR^a^ [kg COD/(m^3^∙day)]	References
Pre-hydrolysed brewers’ spent grain	EGSB	35	0.49 gCOD-CH_4_/(gVSS·day)	10	[36]
Cellulose powder	Batch digestion assays	35	0.18 gCOD-CH_4_/(gVS·day)	N.A.	[37]
Pig manure and rice straw	Batch digestion assays	35	1.31 gCOD-CH_4_/(gVSS·day)	N.A.	[38]
Pig manure and rice straw	Batch digestion assays	55	1.38 gCOD-CH_4_/(gVSS·day)	N.A.	[38]
Corn straw	Full-scale CSTR	37	0.2 gCOD-CH_4_/(gVSS·day)	1.2^b^	[39]
Agro-industrial wastes	Batch digestion assays	55	0.13 gCOD-CH_4_/(gVS·day)	N.A.	[40]
FSF	SBR Digester	35	0.1 gCOD-CH_4_/(gVS·day)	2.5	This study
FSF	SBR Digester	55	0.6 gCOD-CH_4_/(gVS·day)	5.5	This study

*N.A.* data not available

^a^The same period when the SMA of biomass was tested

^b^The unit here is kg TS/(m^3^ day)

### Rheology of mesophilic and thermophilic sludge

Rheology of sludge determines to a large extent the mixing in the reactor systems and the contact between bacteria and its substrate. Rheology of sludge is defined by its viscosity which is a function of applied shear rate and shear stress. Sewage sludges are often non-Newtonian fluids, because the shear rate is not linearly proportional to the applied shear stress [14].

**Fig. 5:**
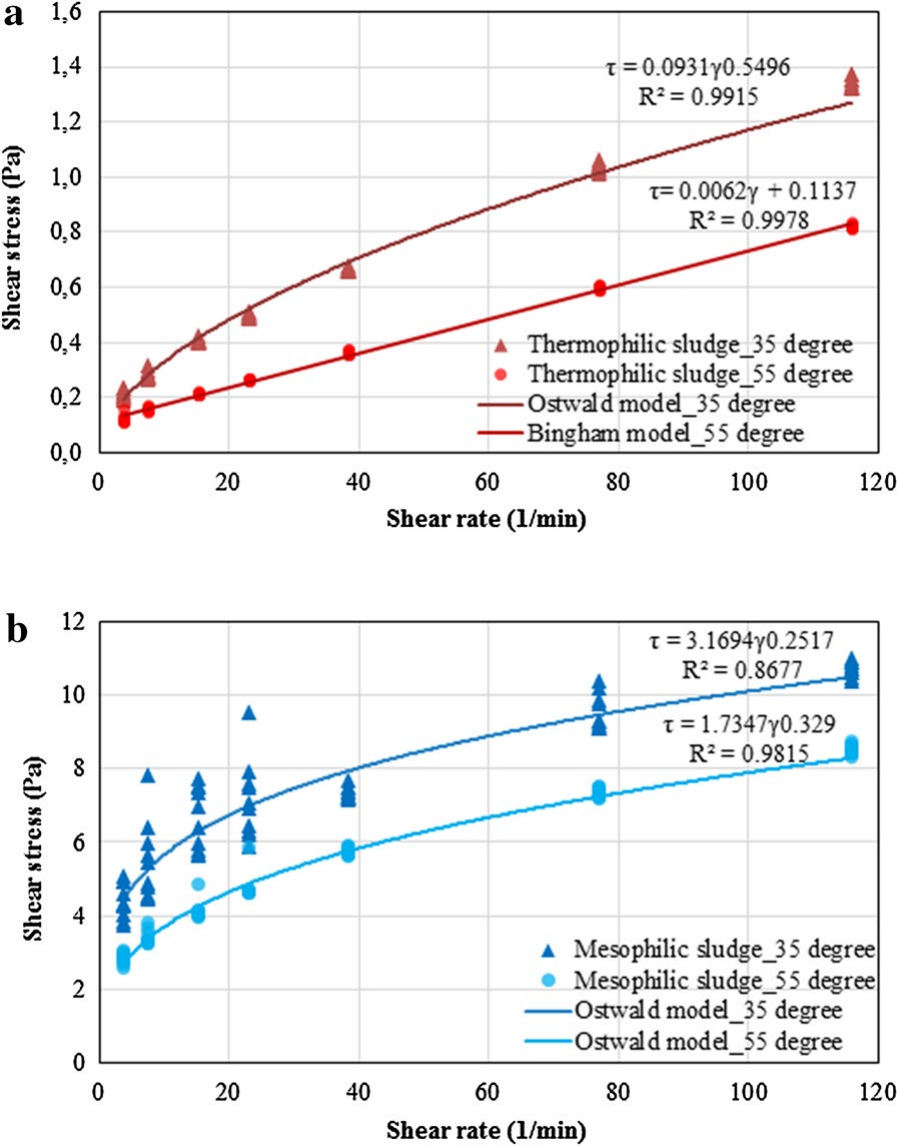
Rheogram of the thermophilic (**a**) and mesophilic (**b**) sludge

Fresh thermophilic and mesophilic sludge were sampled for a rheology test when thermophilic and mesophilic digesters were operated stably (day 526) at an OLR of 5.5 and 2.5 kg COD/(m^3^ day), respectively. The rheogram of both mesophilic and thermophilic sludge was plotted at 35 and 55 °C (Fig. 5). The thermophilic sludge showed a viscoplastic fluids type at 35 °C, while it changed to a Bingham fluids type at 55 °C (Fig. 5). The mesophilic sludge showed a pseudoplastic fluids type at both temperatures (Fig. 5). Both viscoplastic and pseudoplastic fluids have shear thinning effect. An Ostwald–de Waele Power law model (Fig. 5) can be used to describe the rheology behaviour of mesophilic sludge and the thermophilic one that was tested at 35 °C [15]. It is notable that the thermophilic sludge behaved like Newtonian fluids at 55 °C, which meant that the shear thinning effect disappeared when the operational temperature increased from 35 to 55 °C. Meanwhile, the viscosity value of the thermophilic sludge at 55 °C was lower than at 35 °C, which was only 1/18 and 1/34 of the values of mesophilic sludge tested at 55 and 35 °C, respectively, based on the consistency behaviour (0.006 and 0.0931 for thermophilic sludge at 55 and 35 °C, 1.7347 and 3.1694 for mesophilic sludge at 55 and 35 °C) from the Ostwald–de Waele Power law model (Fig. 5). The thermophilic sludge was characterised by a lower viscosity compared to the mesophilic one.

### Microbial population dynamics in the mesophilic reactor

454 pyrosequencing was applied in order to investigate the dynamics of bacterial and archaeal populations at both applied conditions. The changes in microbial communities in the mesophilic reactor before process failure and during recovery were followed closely as well. As expected, the microbial taxa analysed based on a total of about 78,000 sequences showed differences in population between the thermophilic and mesophilic digester over time.

**Fig. 6:**
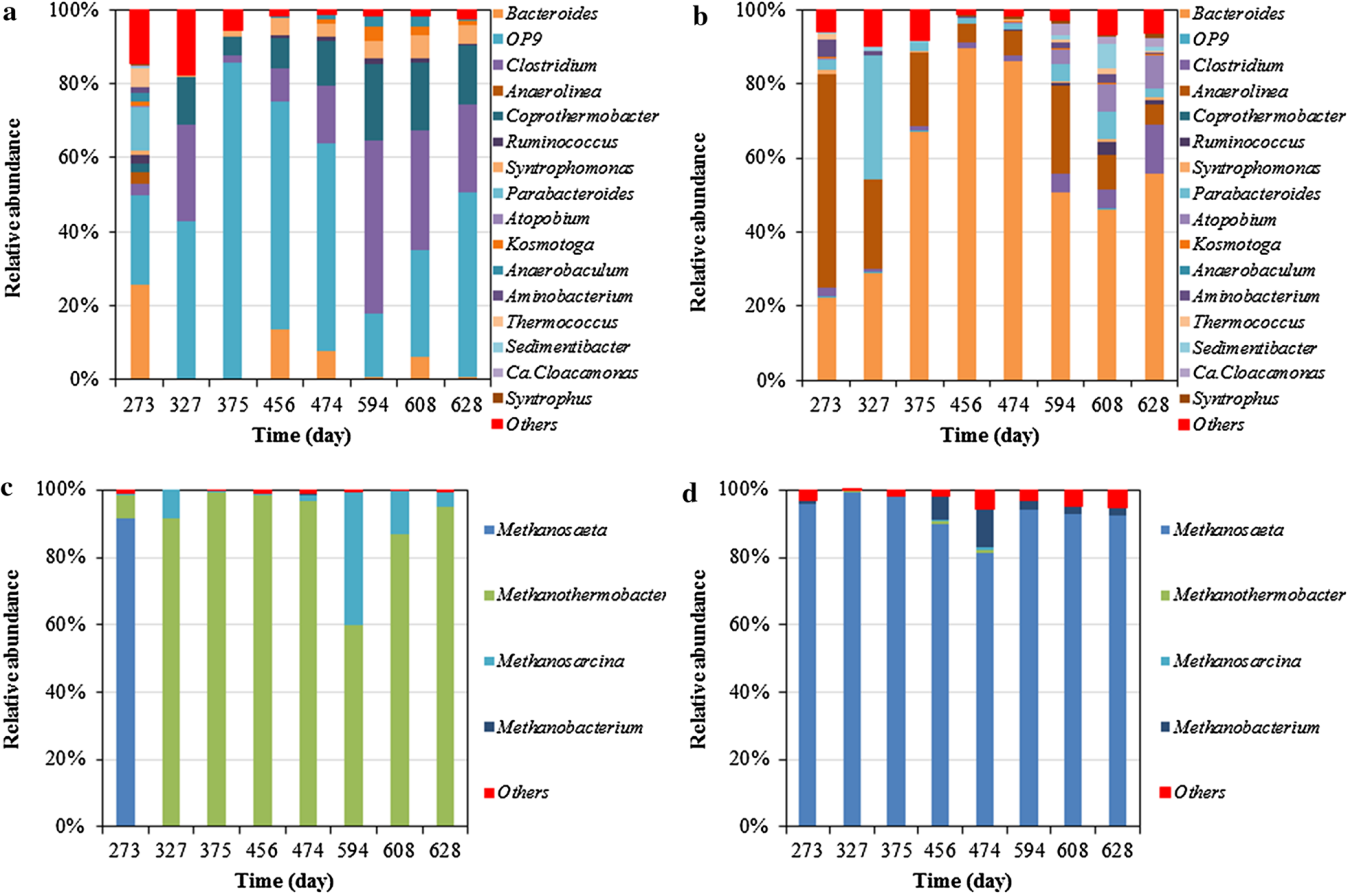
The dynamics and relative abundance of the dominant genera in function of time. **a** bacterial community in the 55 °C digester; **b** bacterial community in the 35 °C digester; **c** methanogenic community in the 55 °C digester; **d** methanogenic community in the 35 °C digester

*Bacteroides* was the sole dominant genus in the mesophilic digester after acclimation (Fig. 6), with variation in its relative abundance from the highest 90 % (sample day 456) to the lowest 46 % (sample day 608). The second predominant genus in the mesophilic digester is *Anaerolinea*, which accounts for 5–24 % in relative abundance. *Parabacteroides* owned 34 % of abundance in the sample of day 327 and 1–12 % in the other samples from the other periods, suggesting its dominant position in the bacterial community as well. *Parabacteroides* are obligatory anaerobic short rods that were frequently discovered in anaerobic environments with capability of producing various acids such as lactic acid, propionic acid, formic acid and acetic acid from carbohydrates [16]. For archaeal lineages in the mesophilic digester, the genus of *Methanosaeta* owned the substantial proportion from 81 to 94 % followed by *Methanobacterium* (2–11 %). *Methanosaeta* is a typical aceticlastic methanogen that prevails in many anaerobic biogas systems [17]. *Methanobacterium* belongs to group of hydrogenotrophic methanogens [18].

About half of the biomass in the mesophilic digester was replaced by stored excess sludge on day 402. Although the exact composition of this excess sludge was unknown, it was anticipated that this microbial community was alike the reactor content before the disturbance at day 393, given the fact that excess sludge was stored without feeding, at room temperature and anaerobic conditions. Every 2 days fresh excess sludge was added to the storage and a new storage container was started every 2 months.

The OLRs of the mesophilic digester were firstly increased to 5.5 kg COD/(m^3^ day) during days of 333–393 and then decreased to 2.5 kg COD/(m^3^ day) from day 393 to day 550 due to VFA accumulation and failure in biogas production. Hereafter, the OLR was increased again, stepwise from 2.5 kg COD/(m^3^ day) to a maximum of 22 kg COD/(m^3^ day). The variation in OLRs directly altered the SRTs of the reactor, which was anticipated to influence the microbial community as well. The abundance of *Bacteroides*, a key genus responsible for hydrolysing and fermenting polysaccharides [19], increased by more than two times when the duration of the batch feeding cycle was shortened from 9 days (day 327, SRT 128 days, OLR 2.5 kg COD/(m^3^ day)) to 2 days (day 375, SRT 64 days, OLR 5.5 kg COD/(m^3^ day)). This sudden increase in *Bacteroides* abundance was concomitant to an observed VFA accumulation from day 394 to 398 (data not shown). The resulting low pH could further favour the growth of *Bacteroides* [20]. Moreover, in the same period, the archaeal community quantity decreased by 60 % from day 327 to 375, while the homo-acetogenic, syntrophic acetate oxidising (SAO) bacteria increased more than three times. SAO bacteria are specialised to oxidise acetate into H_2_ and CO_2_. It was noticed that the thrived growth of the acetate oxidising bacteria could not prevent VFA accumulation, possibly because of an even higher productivity of VFAs from fermenting genera such as *Bacteroides*.

### Microbial population dynamics in the thermophilic reactor

The thermophilic reactor harboured a substantially different microbial community assembly. The predominant bacterial genus altered from OP9 lineage in the samples taken during days 456–474 (relative abundance about 56–62 %) to *Clostridium* in the samples taken after day 594 (relative abundance about 32–47 %), while OP9 was dominant again (relative abundance of 50 %) in the sample of day 628. Members of OP9 have been occasionally observed in anaerobic digestion reactors. For example, they were found to be able to hydrolyse complex carbohydrates such as cellulose under thermophilic conditions [21, 22]. In line with the hydrolytic community, the methanogenic community of the thermophilic digester was also completely different from the mesophilic one. *Methanosaeta* was found to be the absolutely dominant archaeal genus (91 %) until day 273 of the thermophilic digestion, while the hydrogenotrophic methanogen *Methanothermobacter* was only 7 % in relative abundance in the sample of day 273. However, *Methanothermobacter* became the most dominant methanogen afterwards in the thermophilic reactor until the end of operation, having a relative abundance of 60–98 % (Fig. 6).

In the thermophilic reactor, the OLR was stepwise increased from 5.5 kg COD/(m^3^ day) to 22 kg COD/(m^3^ day). *Bacteroides*, OP9 and *Clostridium* were three dominant bacterial genera with total abundance over 50 % under OLR lower than 5.5 kg COD/(m^3^ day) and thus at higher SRTs (samples of day 273 and day 327). By increasing the OLR to 5.5 kg COD/(m^3^ day), OP9 was the sole dominant genera with abundance over 50 % (Fig. 6). When the OLR was further increased to 6.7 kg COD/(m^3^ day) (day 550–608) the dominancy of OP9 was replaced by *Clostridium*, but OP9 again dominated the bacterial community after OLR was increased to 13.5 kg COD/(m^3^ day). *Bacteroides* was dominant once during the start-up period (before day 333) but its abundance decreased and varied in a range between 0.1 and 14 % after the start-up (Fig. 6).

It is interesting that *Methanosarcina*, a group of versatile methanogens that are capable of both aceticlastic methanogenesis and hydrogenotrophic methanogenesis, became dominant (13–39 %) during the same period that *Clostridium* replaced OP9 as the most dominant genera based on the samples of day 594 and 608 (Fig. 6). Then the *Methanosarcina*’s abundance decreased to 4 % on day 628 when *Clostridium* was no longer dominant. *Clostridium* are also versatile microorganisms capable of fermenting cellulose and various carbohydrates into acetate, butyrate carbon dioxide and hydrogen [23, 24]. In line with our observations, it has been reported before that *Clostridium* co-exists with *Methanosarcina* in anaerobic digestion under thermophilic conditions [25].

The combined information from 454-pyrosequencing and real-time quantitative polymerase chain reaction (qPCR) results indicated that a less stable community exists in the mesophilic reactor under the OLRs higher than 5.5 kg COD/(m^3^ day). On the opposite, the thermophilic community was more stable in response to an increasing OLR up to 22 kg COD/(m^3^ day).

### Quantity and activity of microorganisms

**Fig. 7:**
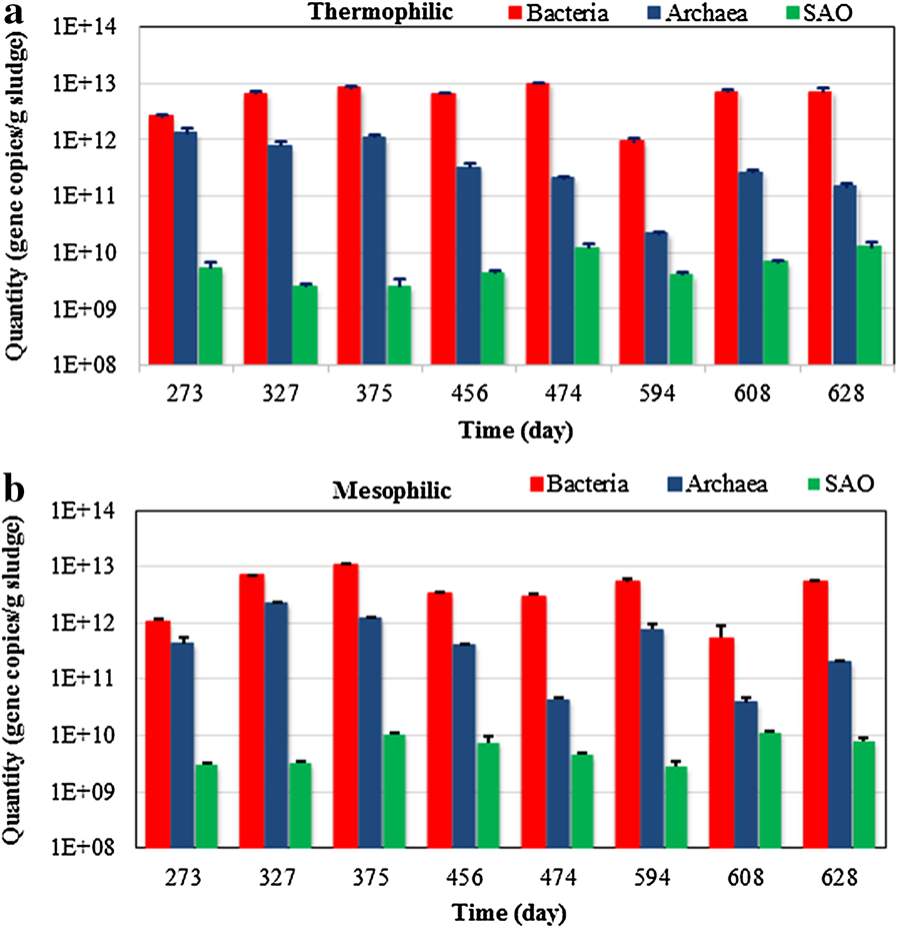
qPCR revealing the quantity of bacteria, archaea and SAO bacteria in the thermophilic reactor (*top*) and mesophilic reactor (*bottom*) at different stages of operating period. Some error bars are too small to be invisible in the above logarithmic co-ordinates

Pyrosequencing data provide taxa information and their relative abundance, but will not give insight into quantities. In this study, qPCR tests based on bacterial, archaeal and SAO bacterial 16S rRNA genes were employed to give insight in specific quantities of bacteria and archaea in general, and SAO bacteria specifically. This analysis showed that the thermophilic reactor harboured marginally higher level of bacterial, archaeal and SAO bacterial 16S rRNA gene copies than the mesophilic reactor (Fig. 7).

For the mesophilic reactor, bacteria had a higher quantity than the archaea and SAO bacteria (Fig. 7). The quantity of bacterial 16S rRNA gene copies had small perturbation around 10^12^–10^13^ gene copies per gram sludge, except for a decrease in the sample of day 608 (Fig. 7), during which period the OLR was increased from 3.4 to 6.7 kg COD/(m^3^ day). The archaeal quantity also fluctuated within the magnitude from 10^10^ to 10^11^ gene copies per gram sludge. The changing trend of archaeal amount was similar to that of bacteria. The SAO bacterial quantity was considerably lower than the other two and fluctuated between 10^9^ and 10^10^ gene copies per gram sludge. It is notable that the concentrations of both bacterial and archaeal gene copies decreased from day 375 to day 456. This is likely because of the pH drop on day 396 and the re-inoculation of stored mesophilic biomass on day 402.

For the thermophilic digester, the quantity distribution was quite similar to the mesophilic reactor, with bacteria having the largest quantity. SAO bacterial amount was stable around 8 × 10^9^ gene copies per gram sludge, while the bacterial and archaeal quantity substantially dropped from day 474 to 594, which coincided to a sudden shift in the dominant bacteria from OP9 to Clostridium. Seasonal differences, changing the raw FSF characterises might have contributed to this change in microbial community. The archaeal quantity varied nearly two magnitudes between the samples taken from day 327 and day 628.

Variation in community structure and microorganisms quantity was observed in both the mesophilic and thermophilic digester; however, the thermophilic digester performed more stably and robust than the mesophilic one. Considering the high percentage of cellulose inside FSF, there likely was more hydrolytic enzymes generated by the thermophilic sludge than by the mesophilic one. The protein analysis by sodium dodecyl sulphate–polyacrylamide gel electrophoresis (SDS-PAGE) and Coomassie staining showed that the thermophilic sludge contained more protein than the mesophilic sludge (*p* < 0.01, Student test) (Additional file 1: Table S2, Figure S1). The protein concentration of the mesophilic sludge fluctuated within the range of 14–72 μg/mL without an obvious trend, while the protein concentration of the thermophilic sludge almost increased by three times from day 356 (OLR = 5.5 kg COD/(m^3^ day)) to day 624 (OLR = 10 kg COD/(m^3^ day)).

The quantity of microorganisms and their released protein together can indicate efficiency of an anaerobic digester, which is normally, from an engineering retrospective, a direct response to operational parameter(s). From an engineering retrospective, the food to mass ratio (F/M ratio) or OLR, highly determines the design and applicability of a process. The higher the applicable OLR, the compacter the reactor and thus the more biogas can be produced per reactor volume. However, overloading a digester could lead to process instability due to accumulated VFA and decreased pH (as on day 396). In our study, both reactors were applied with varying OLRs, which has been comprehensively proven as a driving force altering digesters’ microbiome [26, 27]. Our results indicated that the applicable OLRs to a mesophilic digester was usually lower than that to a thermophilic process, possibly due to a limitation of methanogenic capacity (Fig. 4) and an instable methanogenic community (Fig. 6d) under mesophilic conditions. However, a higher OLR at 22 kg COD/(m^3^∙day) could be applied to the thermophilic digester when hydrolytic bacteria were abundant (Fig. 6a) and large amount of hydrolytic enzymes could be observed (Additional file 1: Table S2, Figure S1).

### Microbial diversity

**Fig. 8:**
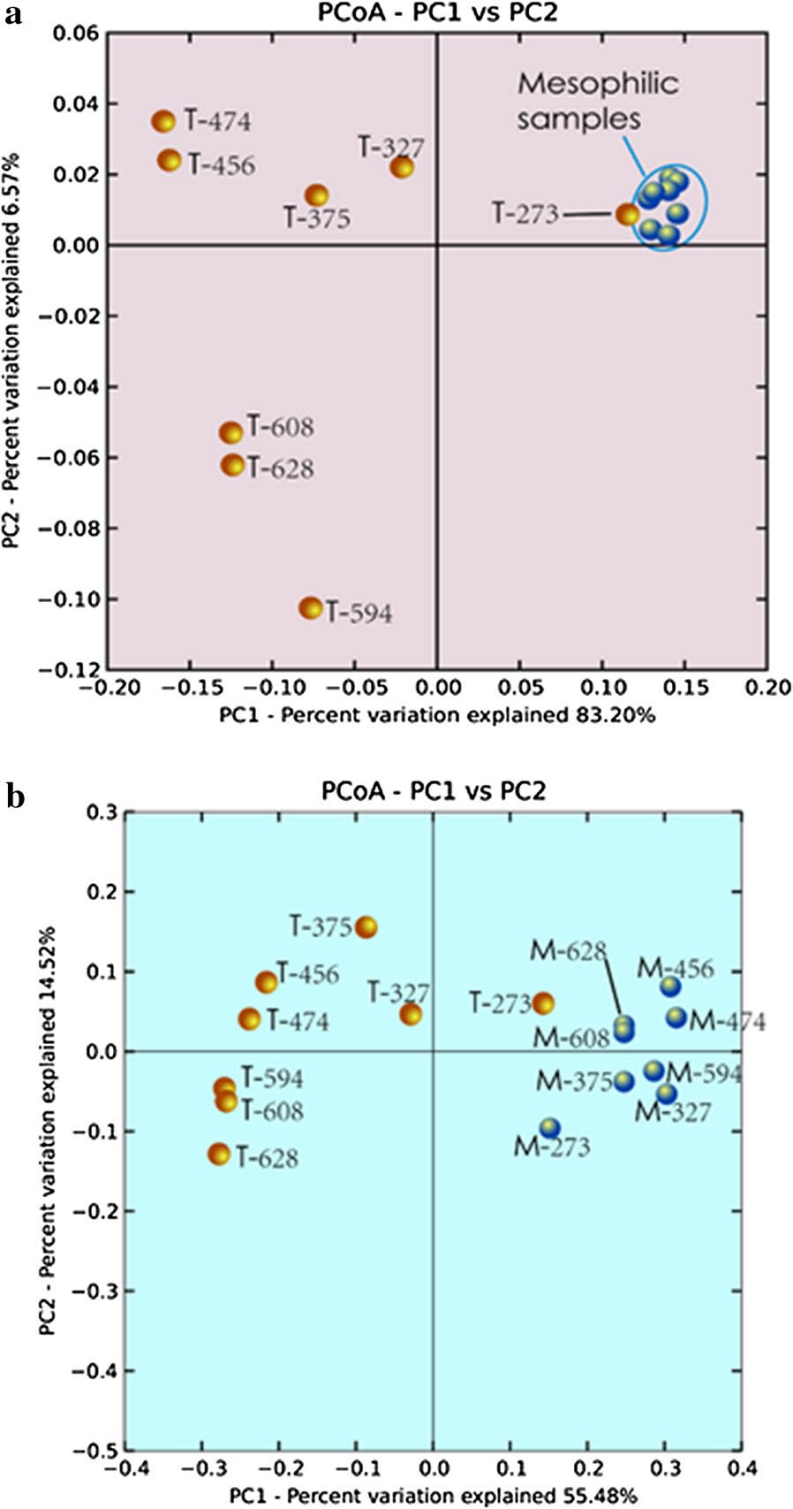
Principal co-ordinate analysis (PCoA) of bacterial (**a**) and archaeal (**b**) communities of the biomass that were sampled at different times from the thermophilic (marked with T) and mesophilic (marked with M) reactors

Both α- and β-diversity were analysed based on 454-pyrosequencing raw data. The α-diversity is defined as the diversity of organisms in one sample or environment and the β-diversity is the difference in diversities across samples or environments. We employed phylogenetic distance (PD), the observed number of operational taxonomic units (OTUs), Chao1 and Shannon index to fully characterise the α-diversity of each community, all of which are commonly used in recent years [28]. For β-diversity, UniFrac method is considered to be very useful in revealing biologically meaningful patterns [28] and so was applied in this study (Fig. 8).

Our results showed that the bacterial communities, for both thermophilic and mesophilic digesters, owned higher richness and evenness than the archaeal communities (Additional file 1: Figure S2). Both thermophilic and mesophilic bacterial community shared similar level of evenness (Shannon index, Additional file 1: Figure S2a, S2b), while the archaeal community of the mesophilic reactor (Shannon index, Additional file 1: Figure S2d) showed a lower level of evenness than the thermophilic reactor (Shannon index, Additional file 1: Figure S2c). The observed species number of the thermophilic community (both bacterial, Additional file 1: Figure S2a, and archaeal, Additional file 1: Figure S2c) was more stable than the mesophilic one (Additional file 1: Figure S2b, S2d). The β-diversity results proved that there was a stable archaeal community in the mesophilic digester as all blue dots were close to each other (Fig. 8a). Also, the bacterial community in the mesophilic digester was more stable compared to the thermophilic reactor (Fig. 8b). In other words, the dynamics of the thermophilic community was more intense than that of the mesophilic community.

Vanwonterghem et al. [17] suggested that a deterministic process was important to microbial community dynamics in long-term operated anaerobic digesters, when a selective pressure imposed by the operational conditions existed [17]. In this study, the thermophilic reactor reveals a clear evolution of the bacterial community (Fig. 8a), whereas the archaeal community in the same reactor showed a weaker pattern of evolution. The performance of the thermophilic reactor was primarily disclosed by the structure and functionality of bacterial community. Two important facts played a role: (1) the hydrolytic populations were consistently and strongly adapted during the OLR increase and (2) an efficient syntrophic relationship was eventually established at high OLRs. From this point of view, our study indicated that a deterministic process also guided microbial community dynamics in a thermophilic digester. For the mesophilic reactor, it is difficult to conclude a deterministic or a stochastic process of population dynamics because of the re-inoculation in the middle stage of the operation.

## Conclusions

FSF as a new type of concentrated substrate sequestered from raw municipal sewage contains high fraction of cellulosic fibres originating mainly from toilet paper, hence FSF is rich in energy. Our study demonstrates that FSF from a domestic sewage treatment plant that has 23 % dry solids content can be digested either under thermophilic (55 °C) or mesophilic (35 °C) conditions. The long-term adapted microbial communities at 55 and 35 °C were distinctly different in composition and population dynamics. In the thermophilic community, OP9, *Clostridium* and *Methanothermobacter* while in the mesophilic community *Bacteroides* and *Methanosaeta* were the dominant species. Eventually, the thermophilic digester produced biogas stably at an extreme loading rate of 22 kg COD/(m^3^ day). In addition, the thermophilic sludge had a considerably lower viscosity than the mesophilic sludge. Results clearly show the high rate potentials of thermophilic conditions for the digestion of sewage FSF under extreme loading conditions.

## Methods

### Digester

Four water-jacketed laboratory mixed digesters with a working volume of 8 L were used in duplicate to conduct the digestion of FSF under both thermophilic and mesophilic conditions for more than 650 days at 55 and 35 °C, respectively. The reactors were continuously mixed by stirring (60-80RPM, Maxon motor Benelux B.V., Switzerland) to create a homogenised matrix. The system was equipped with a pH and temperature probe (CPS41D, Endress + Hauser B.V., Switzerland) and an online biogas measuring device (RITTER MilliGascounter MGC-1 PMMA, Germany). The temperature was controlled by circulating water from a programmable water bath (TC16, PMT TAMSON, the Netherlands). The pH, temperature, biogas flow rate were continuously monitored using Labview software.

### Substrate

A rotating belt filter (Salsnes Filter, Norway) equipped with a 350-µm pore size fine sieve was operated to treat the screened (mesh size 6 mm) sewage at WWTP Blaricum, the Netherlands (plant size: 30,000 pe, maximum hydraulic capacity 1600 m^3^/h). The fine sieved fraction (FSF) coming from this sieve was collected once every 4 months and stored at 4 °C prior to use. The FSF contained mainly paper fibres, some sand, hair, leaves and undefined materials. The main physicochemical characteristics of the different used FSFs batches were determined after every sampling event at WWTP Blaricum and prior to use [3]. This heterogenetic appearance of FSF (Additional file 1: Figure S3) was mainly due to seasonal fluctuations (e.g. more leaves in the FSF in autumn), functioning of the fine sieve system, FSF storage time and temperature in the on-site container. The maximum period that the FSF was stored in the container was 2 weeks and the temperature varied from maximum 25 °C in summer to minimum 0 °C in winter. FSF was fed manually and batch wise in a way that first the corresponding mass to be fed was extracted from the reactor where after the reactor was fed with FSF.

### Inoculum

It would be difficult and inefficient to inoculate both thermophilic and mesophilic digester using the same seed sludge, hence, two origins of seed sludge were chosen in this study. In the first stage (start-up of the experiment), the thermophilic inoculum was obtained from a plug flow dry anaerobic composting (DRANCO, OWS, Brecht, Belgium) digester [29], operated at a solids retention time (SRT) of 15 days and treating mainly vegetable, fruit and yard wastes with a dry matter content of about 35 % and a heterogeneous appearance. The thermophilic inoculum was sieved (4 mm mesh) prior to use. Mesophilic inoculum was taken from an anaerobic digester of a WWTP (Harnaschpolder, Delft, the Netherlands) that treats both primary and secondary sludge with a maximum solid content of 5 % and which was operated at an SRT of 22 days. In the second stage of this study, both adapted thermophilic and mesophilic sludges were taken directly from the FSF-fed laboratory scale anaerobic digesters that were operated at a dry solids content in the range of 4–7 %. Due to reactor instability half of the biomass (about 120 gVS) in the mesophilic digester was replaced on day 402 by excess sludge that was collected from the same reactor. This excess sludge was collected each feeding period and stored for a maximum of 2 months at room temperature and no feeding was applied. The biogas produced in the excess sludge container was released every 2 days.

### Analytical methods

Total solids (TS) and volatile solids (VS) were determined on weight base (g/kg) according to the standard methods for the examination of water and wastewater [30]. Chemical oxygen demand (COD, 500–10,000 mg/L) was measured spectrophotometrically using photometric cell tests (Merck, Germany). All analyses were done in triplicate.

Volatile fatty acids (VFAs) were quantified by Gas Chromatograph (GC, Agilent Technology 7890A), using a flame ionisation detector (FID) and a capillary column type HP-FFAP Polyethylene Glycol (25 m × 320 μm × 0.5 μm) with helium as the carrier gas at a total flow of 67 ml/min and a split ratio of 25:1. The GC oven temperature was programmed to increase from 80 °C to 180 °C in 10.5 min. The temperatures of injector and detector were 80 and 240 °C, respectively, and the injected volume was 1 μl. Prior to GC analysis 10 ml of digested samples was first centrifuged at 15,000 rpm for about 15–20 min. Then the supernatant was filtrated over 0.45 µm filter paper. The filtrated liquid was diluted 2 and 3 times with pentanol as internal solution (300 ppm) for mesophilic and thermophilic digestion samples, respectively. Finally, 10 µL of formic acid (purity >99 %) was added into the 1.5 mL vials.

Gas composition (CH_4_, CO_2_) was measured by using a GC [Agilent 7890A, with Agilent 19095P-MS6 + 19095P-UO4 (60 m × 530 μm × 20 μm)] equipped with a thermal conductivity detector (TCD, reactor experiments). Helium was used as a carrier gas with a split flow of 10 ml/min and operation conditions were: Oven 100 °C (45 °C for 6 min then 25 °C/min to 100 °C for 1.8 min), detector 200 °C and injection port 45 °C. The biogas volume was measured by Milligascounter. Biogas results were then converted to standard temperature and pressure conditions (T = 0 °C and P = 1 atm).

### Specific methanogenic activity (SMA)

Specific methanogenic activity (SMA) was used to determine the conversion rate of acetate into CH_4_ in the anaerobic system. In this study, the SMA of the mesophilic and thermophilic sludge was determined using an Automated Methane Potential Test System (AMPTS II, Bioprocess Control, Lund, Sweden). The SMA was conducted using sodium acetate COD (2 g/L) as substrate and supplemented by a medium consisting of a mixture of macronutrients, trace elements and phosphate buffer solution [31]. The inoculum amount was determined by setting an inoculum VS to substrate COD ratio on weight base (I/S) of 2:1. SMA was calculated by the slope of the accumulated methane production curve (mL/day) divided by the grams of VS introduced in the bottle (inoculum). The final values were expressed in gCH_4_-COD/(gVS. day). Experiments were conducted in triplicate.

### Anaerobic biodegradability

Anaerobic biodegradability (AnBD) was assessed as the experimental ultimate methane production (expressed in COD) over the initial tCOD of the substrate [32].

Giving the conversion 1 CH_4_ + 2O_2_ → CO_2_ + 2H_2_O, 1 g COD equals 350 mL of CH_4_ at 273 K and 1 bar. It is noted that this theoretical approach does not take into account the needs for bacterial cell growth and their maintenance, which has been reported typically 5–10 % of organic material degraded [31], meaning that not all the COD is methanised. Moreover, during bioconversion, non-methanised biodegradable or non-biodegradable intermediates may occur, lowering the actual methane yield of the substrate.

### Reactor operation

Four water-jacketed laboratory mixed digesters with a working volume of 8 L were used in duplicate to conduct the digestion of FSF under both thermophilic and mesophilic conditions for more than 650 days at 55 and 35 °C, respectively. The first 333 days of the experiment were mainly used to adapt the sludges to the FSF, especially the thermophilic sludge [3]. At the same period, several biomethane potential (BMP) tests and SMA tests were conducted to monitor the conversion capacity and activity of the biomass at both conditions. In the first stage of the study (day 333–393), it was decided to operate both thermophilic and mesophilic digesters at an organic loading rate (OLR) of 5.5 kg COD/(m^3^ day), applying an FSF COD and TS of 350 ± 15 g/kg and of 25 %, respectively. In the second stage of this study (day 393 till day 656), the OLR was increased stepwise from 2.5 kg COD/(m^3^ day) and 5.5 kg COD/(m^3^ day) to a maximum of 22 kg COD/(m^3^ day) for both the mesophilic and thermophilic digesters, resulting in decreasing solid retention times (SRT). Table 1 shows the digesters’ operation performance within the extended research period and batch cycle duration of 2 days.

### Environmental scan electron microscopy and energy dispersive X-ray element analysis (ESEM-EDX)

The samples for ESEM and EDX analysis were collected from both mesophilic and thermophilic digesters that were operated at an OLR of 2.5 and 5.5 kg COD/(m^3^ day), respectively. The structure of the substrate before degradation was studied using an ESEM equipped with an EDX elemental analysis system. Raw FSF and sludge samples were pretreated immediately after collection. Triplicated samples were washed for three times using phosphate-buffered saline (PBS) (Sigma Aldrich). Then the samples were air dried and ready for analysis. Before ESEM analysis, the samples were mounted on a 1-cm^2^ metal support and kept in place with adhesive tape and observed with a Philips XL30 Series ESEM. An EDAM 3 EDS system (SUTW 3.3 EDX window and 128.0 eV EDX resolution) was applied to analyse the key elements.

### Viscosity analysis

Fresh thermophilic and mesophilic sludge were sampled for rheology tests on day 526 when the operating OLRs were 5.5 and 2.5 kg COD/(m^3^ day), respectively. Rheological characteristics were determined with a universal dynamic rheometer (Paar Physica UDS 200, Stuttgart, Germany) equipped with a waterbath for temperature adjustment. The software US200/32 (V2.30) was applied for programming and data logging. For this purpose, it was attempted to conduct the test at two different temperatures, i.e. 35 and 55 °C, for both types of sludge.

### DNA extraction

Fresh biomass samples were washed with 1 × PBS and then centrifuged under 7000×*g* for 7 min. The supernatant was removed and the pellet was washed by PBS for a second time and centrifuged under 17,000×*g* for 20 min. The supernatant was removed and the pellet was stored (less than 1 month) at −25 °C for DNA extraction. DNA extraction was performed using the MoBio UltraClean microbial DNA isolation kit (MoBio Laboratories, CA, USA). A minor modification of the manufacturer’s protocol was that twice bead-beating (5 min) and heating (5 min) were applied in sequence in order to enhance the lysis of microbial cells. DNA isolation was confirmed by agarose gel electrophoresis. The quality of DNA was verified by Nanodrop 1000 (Thermo Scientific, Waltham, MA, USA).

## 454 Pyrosequencing

The amplification and sequencing of the 16S rDNA gene was performed by Research and Testing Laboratory (Lubbock, TX, USA) with following primers: (1) U515F (5′-GTG YCA GCM GCC GCG GTA A-3′) and U1071R (5′-GAR CTG RCG RCR RCC ATG CA-3′) [33] were used for bacteria and archaea with a high coverage over 90 % for each domain; (2) Arch341F (5′-CCC TAY GGG GYG CAS CAG-3′) and Arch958R (5′-YCC GGC GTT GAM TCC AAT T-3′) were used for archaea. The pyrosequencing was done using a Roche 454 GS-FLX system (454 Life Science, Branford, CT, USA) with titanium chemistry.

### Post analysis of pyrosequencing data

The post analysis of pyrosequencing data was performed by combining different programs from the Quantitative insights into microbial ecology (QIIME) pipeline, version 1.6.0 [34].

### Real-time qPCR

Real-time qPCR was performed using an ABI 7500 instrument (Foster City, CA, USA) with three primer sets, including Bac516-F-Bac805-R (for all bacteria), ARC787-F-ARC1059-R for all archaea, FTHFS-F-FTHFS-R for syntrophic acetate-

oxidising bacteria [35]. qPCR amplification was done in a 20-μL reaction volume. Each reaction tube contained 10 μL 2 × SGExcel FastSYBR Mixture (With ROX, Sangong Biotech, Shanghai, China), 8.6 μL dH_2_O, 0.2 μL of each forward and reverse primer (1 pmol/μL), and 1 μL of DNA template. Molecular grade water was used as a negative control. Triplicate PCR reactions were carried out for all samples and negative controls. The thermal cycling program consisted of 2 min at 50 °C, 1 min at 95 °C, followed by 40 cycles of 10 s at 95 °C, 35 s at X  °C (X = 56 for Bac516-F/Bac805-R, X = 61 for ARC787-F/ARC1059-R, X = 55 for FTHFS-F/FTHFS-R). Finally, a melting curve analysis was performed for verifying the specificity of PCR products; denaturation of 1 min at 95 °C, cooling of 1 min at 55 °C and then heating till 95 °C again, at a rate of 0.5 °C per cycle. The standard curves for the above primer sets were constructed using all strains of the samples. The target 16S rDNA gene sequences were amplified from each strain by PCR with the corresponding primer sets and cloned into pGEM-T Easy vectors (Sangong Biotech, Shanghai, China).

For each plasmid, a tenfold serial dilution series ranging from 10^10^ to 10^4^ copies/mL was generated. The slopes of the plasmid standard curves were between −4.411 and −2.955, with a mean value about −3.313. The threshold cycle (*C*_T_) values determined were plotted against the logarithm of their initial copy concentrations. All standard plasmids and 16S rDNA samples were amplified in triplicate.

## Additional file


10.1186/s13068-015-0355-3 Supplementary materials.

## Abbreviations

AD: anaerobic digestion
AMPTS: automatic methane potential test system
AnBD: anaerobic biodegradability
BMP: biological methane potential
COD: chemical oxygen demand
CSTR: continuous-stirred tank reactor
EDX: energy dispersive X-ray
EGSB: expanded granular sludge bed
ESEM: environmental scan electron microscopy
FSF: fine sieved fraction
GC: gas chromatograph
I/S: inoculum to substrate ratio
OLR: organic loading rate
OUT: operational taxonomic unit
PCoA: principal co-ordinate analysis
PD: phylogenetic distance
qPCR: quantitative polymerase chain reaction
SAO: syntrophic acetate oxidising
SBR: sequencing batch reactor
SDS-PAGE: sodium dodecyl sulphate–polyacrylamide gel electrophoresis
SMA: specific methanogenic activity
SRT: solid retention time
STP: sewage treatment plant
tCOD: total chemical oxygen demand
TS: total solids
VFA: volatile fatty acid
VS: volatile solids
