# Supplementary material for: Microbial population dynamics during long-term sludge adaptation of thermophilic and mesophilic sequencing batch digesters treating sewage fine sieved fraction at varying organic loading rates
Source: Biotechnol Biofuels. 2015 Oct 21;8:171. doi: 10.1186/s13068-015-0355-3 (PMC4618146; doi:10.1186/s13068-015-0355-3)
Supplement: Supplementary file 1 — 10.1186/s13068-015-0355-3 Supplementary materials. [file 13068_2015_355_MOESM1_ESM.docx]

**Supplementary materials**

**Microbial population dynamics during long term sludge adaptation of thermophilic and mesophilic sequencing batch digesters treating sewage fine sieved fraction at varying organic loading rates**

Dara S.M. Ghasimi*, Yu Tao, Merle de Kreuk, Marcel H. Zandvoort, Jules B. van Lier

***Corresponding author**:

Dara S.M. Ghasimi (E-mail: [S.M.D.Ghasimi@tudelft.nl](mailto:S.M.D.Ghasimi@tudelft.nl); Tel: +31 (0)616694522

**Contents**

**Supplementary methods**

**Supplementary Tables**

**Supplementary Figures**

# Supplementary methods

## SDS–polyacrylamide gel electrophoresis

Sodium dodecyl sulphate polyacrylamide gel electrophoresis (SDS-PAGE) was performed according to the NuPage® electrophoresis system instructions (InvitrogenTM Life Technologies, Carlsbad, CA, USA). Protein separation was performed using a precast NuPAGE 4-12% Bis-Tris Gel (Novex® Life Technologies, CA, USA). Protein concentrations were determined with a Bradford assay using bovine serum albumin (BSA) as a reference. Each sample was mixed with lithium dodecyl sulphate (LDS) sample buffer with an additional 5 mM DTT and heated at 95 oC for 5 minutes. After heating, the mixtures were centrifuged at 13,300 g for 5 minutes and the supernatant was immediately applied to the gel. For samples 1 to 8, 25 µl sample was loaded, for samples 9 to 17, 5 µl was loaded. The electrophoresis was done in MES SDS running buffer (Novex® Life Technologies, CA, USA) at a constant voltage of 200 V for 35 minutes. Gels were subsequently stained with the Colloidal Blue Staining Kit (Novex® Life Technologies, Carlsbad, CA, USA) according to the manufacturer’s instructions.

# Supplementary Tables

## Table S1 ESEM-EDX results revealing element content in the targeting spots of Fig. 1f and 1i

| **Elements** | Fig. 1f | | Fig. 1i | |
| --- | --- | --- | --- | --- |
|  | **Weight (%)** | **Atom (%)** | **Weight (%)** | **Atom (%)** |
| C | 71.88 | 79.77 | 64.42 | 77.04 |
| O | 19.99 | 16.65 | 15.50 | 13.91 |
| Na | 2.10 | 1.22 | 3.26 | 2.04 |
| Al | 0.37 | 0.18 | 0 | 0 |
| Si | 0.94 | 0.45 | 0.40 | 0.21 |
| P | 0.69 | 0.30 | 0.78 | 0.40 |
| S | 0.75 | 0.31 | 1.79 | 0.83 |
| Cl | 1.67 | 0.63 | 1.85 | 0.83 |
| Ca | 1.16 | 0.39 | 1.95 | 0.70 |
| Fe | 0.45 | 0.11 | 0.56 | 0.14 |
| Mg | 0 | 0 | 0.27 | 0.16 |
| K | 0 | 0 | 0.27 | 0.10 |
| Total | 100.00 | 100.00 | 100.00 | 100.00 |

## Table S2 Samples for SDS-PAGE & coommassie staining and protein concentrations for mesophilic and thermophilic sludges

| Sample No. | Day | OLR kg COD/(m^3^∙day) | Protein concentration  μg /mL | |
| --- | --- | --- | --- | --- |
| Mesophilic sludge | | | |  |
| 1 | 273 | Adaptation period | 64.4 | |
| 2 | 306 | Adaptation period | 52.4 | |
| 3 | 356 | 5.5 | 34.4 | |
| 4 | 540 | 2.5 | 52.4 | |
| 5 | 571 | 3.4 | 14.4 | |
| 6 | 600 | 3.4 | 48.4 | |
| 7 | 624 | 10 | 72.4 | |
| Thermophilic sludge | | | |  |
| 8 | 273 | Adaptation period | 182.4 | |
| 9 | 306 | Adaptation period | 152.4 | |
| 10 | 356 | 5.5 | 148.4 | |
| 11 | 540 | 5.5 | 238.4 | |
| 12 | 571 | 6.7 | 256.4 | |
| 13 | 600 | 6.7 | 292.4 | |
| 14 | 624 | 10 | 434.4 | |

# Captions of supplementary figures

**Figure S1** SDS-PAGE & coommassie staining of mesophilic (1-7) and thermophilic (8-14) sludge that were sampled at different time of operation (Table S2).

**Figure S2** Alpha-diversity of bacterial and archaeal community at different stages in the thermophilic and mesophilic reactors. a, bacterial community in the thermophilic digester; b, bacterial community in the mesophilic digester; c, archaeal community in the thermophilic digester; d, archaeal community in the mesophilic digester.

**Figure S3** Photos of raw FSF at different sampling time: dry weather (left) and wet weather (right). The heterogenetic appearance of the raw FSF was because of several reasons, such as seasonal fluctuations (e.g. more leaves in the FSF in autumn), changes of functioning of the fine sieve system, storage time of FSF, and temperature in the on-site container storing FSF.


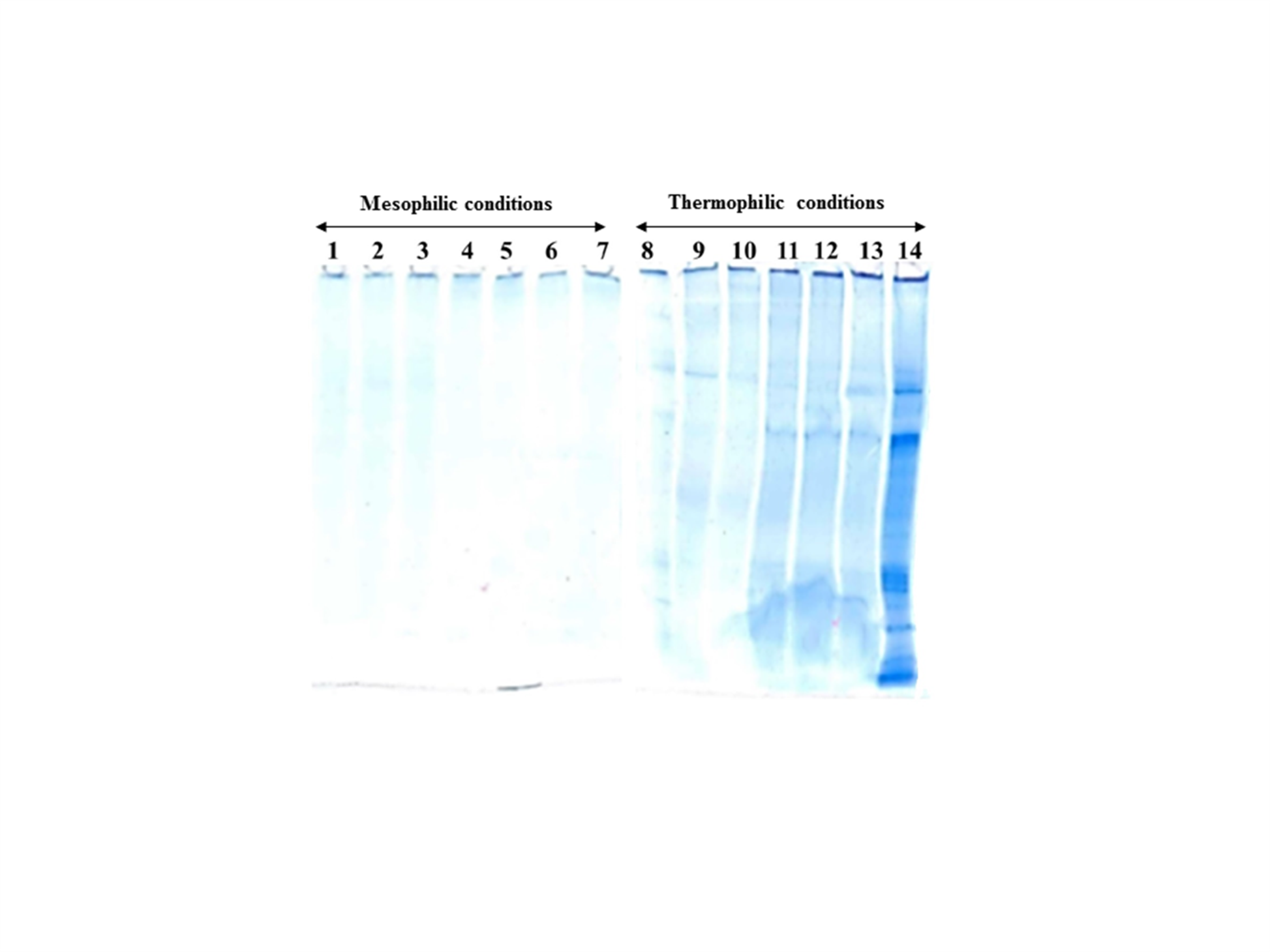


## **Figure S1**


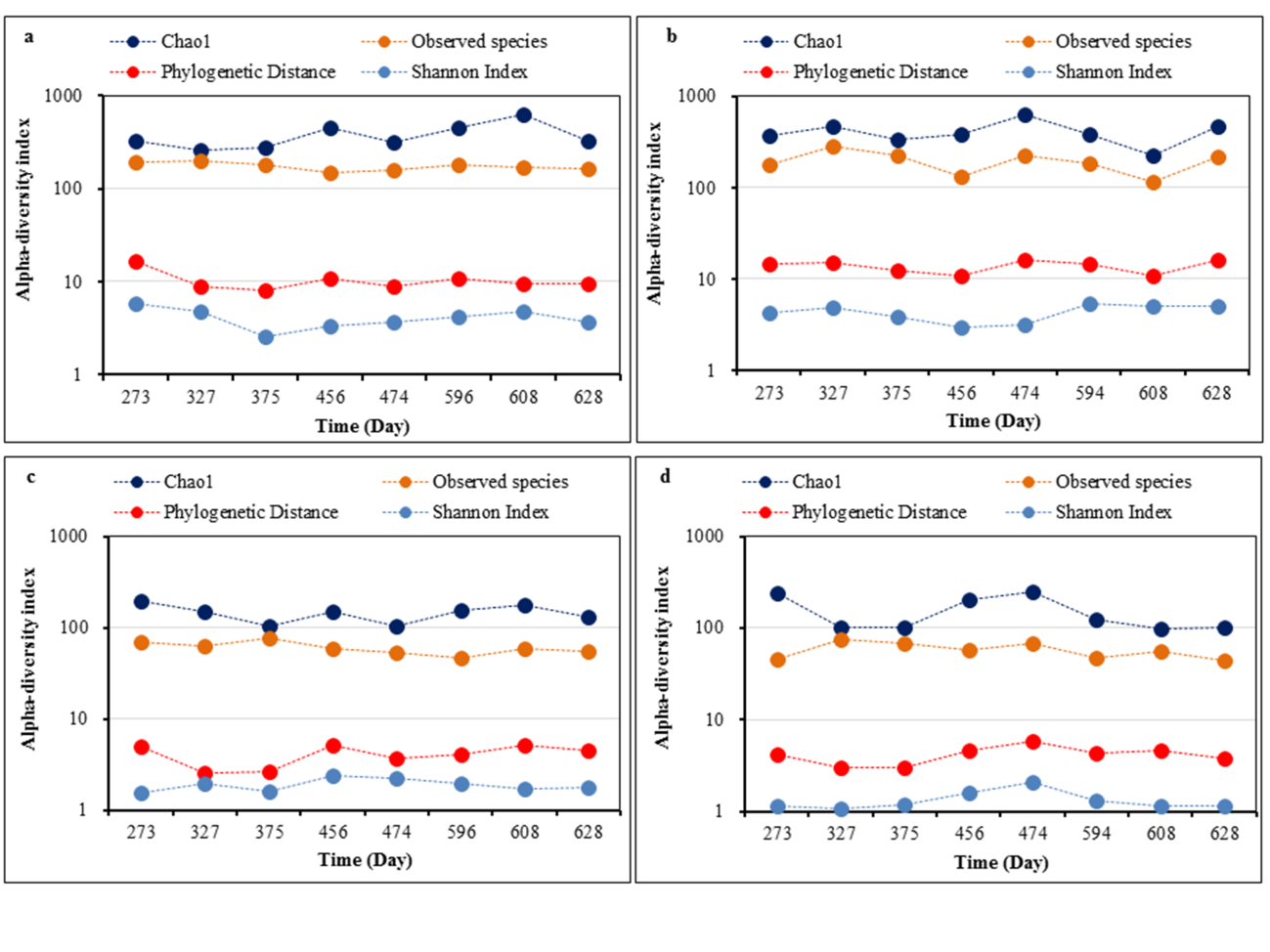


## **Figure S2**


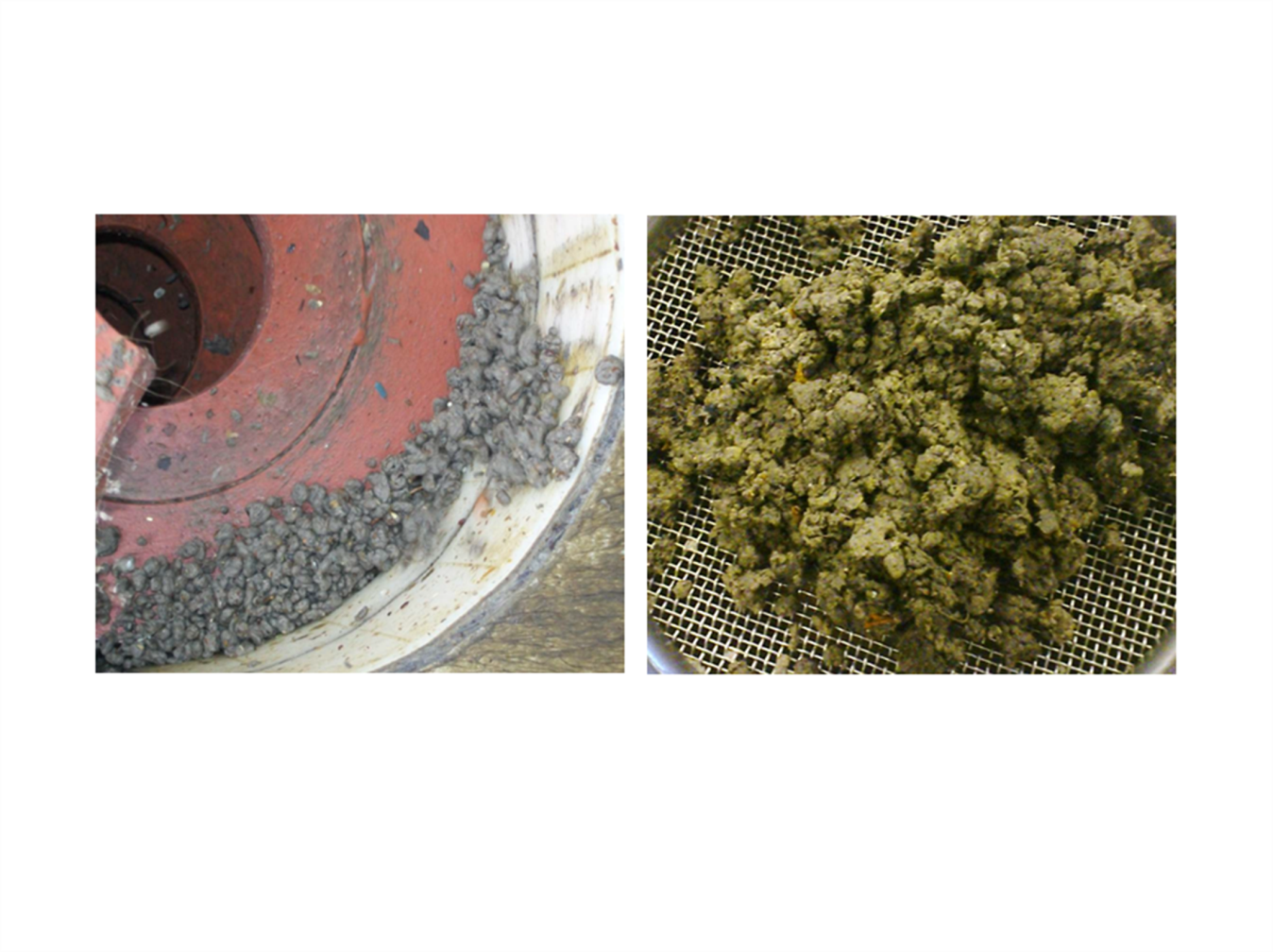


## **Figure S3**
